# Hydatid disease beyond the liver: An unusual multi-organ presentation in a 2-year-old girl

**DOI:** 10.1016/j.radcr.2025.07.015

**Published:** 2025-08-08

**Authors:** Nabil Ziani, Mounir Salek, Abir Saalaoui, Abderrahmane Ibenyahia, Houssam Rajad, Houcine Ben Salah, Meryem Amghar, Nour El Houda Laffinti, Karim Baayoud, Soukaina Wakrim

**Affiliations:** a Radiology Department, University Hospital of Souss Massa, Agadir, Morocco; b Faculty of Medicine and Pharmacy of Agadir, Ibn Zohr University, Agadir, Morocco; c Neurosurgery Department, University Hospital of Souss Massa, Agadir, Morocco

**Keywords:** Hydatid disease, Hydatid Cyst, Pediatric, Multimodal imaging, Brain

## Abstract

Hydatid disease is endemic in regions like Morocco, commonly affecting the liver and lungs. In pediatric patients, the condition is uncommon, and multi-organ involvement without hepatic lesions is exceedingly rare. We report the case of a 2-year-old girl diagnosed with hydatid cysts involving the central nervous system, lungs, and right kidney. Notably, imaging studies revealed no hepatic involvement, which is highly unusual for echinococcosis. This case highlights an atypical presentation of pediatric hydatid disease and illustrates the essential role of comprehensive radiologic assessment in endemic areas. Early identification of extrahepatic involvement can guide appropriate management and improve outcomes.

## Introduction

Hydatid disease (cystic echinococcosis) is a parasitic infection caused by the larval stage of *Echinococcus granulosus*, primarily affecting populations in endemic regions such as the Mediterranean, Middle East, South America, Africa, and Asia [1]. The liver is the most commonly involved organ (around 70% of cases), followed by the lungs (20%), with other localizations being rare [1]. Pediatric cases represent a smaller fraction and often pose diagnostic challenges due to atypical presentations and unusual organ involvement [2].

Multiorgan hydatid disease in children is uncommon, and presentations without hepatic involvement are to our knowledge, rarely reported, defying the typical dissemination route where the liver and lungs serve as primary filters [3]. Literature describes isolated cysts in the brain, spleen, kidneys, or muscles, but simultaneous extrahepatic localizations remain atypical [4,5].

This report presents a rare case of disseminated extrahepatic hydatid disease in a 2-year-old girl—confirmed via multimodal imaging and histopathology—highlighting the need to consider non-hepatic cystic echinococcosis in pediatric patients from endemic regions.

## Case presentation

A 2-year and 3-month-old female child, with no history of perinatal complications, presented with a 2-month history of febrile episodes accompanied by tonic-clonic seizures. Neurological examination revealed left-sided hemiparesis and left facial palsy, with no additional symptoms. The patient lives in a rural endemic area, however there was no relevant family history of hydatic disease, nor any contact with livestock animals.

**Fig. 1:**
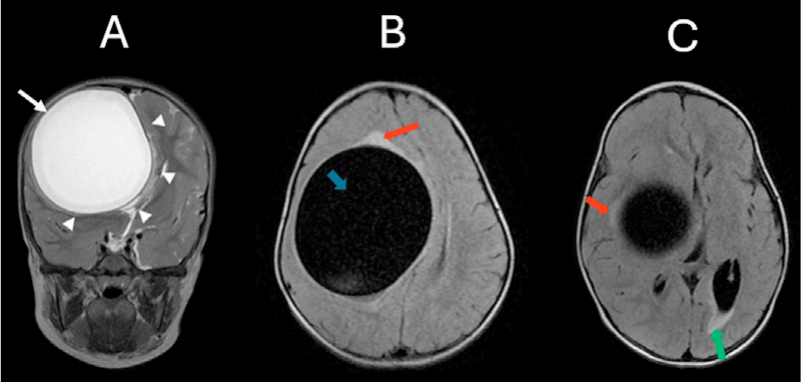
(A) Coronal T2-weighted MRI demonstrates a large, well-defined intra-axial cystic lesion in the right frontoparietal region with homogeneously hyperintense signal content (white arrow). The lesion exerts significant mass effect on the midline structures (white arrowheads). (B & C). Axial T2 FLAIR images show complete suppression of the cystic fluid signal within the lesion (blue arrow). A faint hyperintense pericystic rim is noted (red arrow), suggestive of minimal surrounding edema. Image C additionally reveals periventricular FLAIR hyperintensity, most pronounced along the left occipital horn (green arrow).

**Fig. 2:**
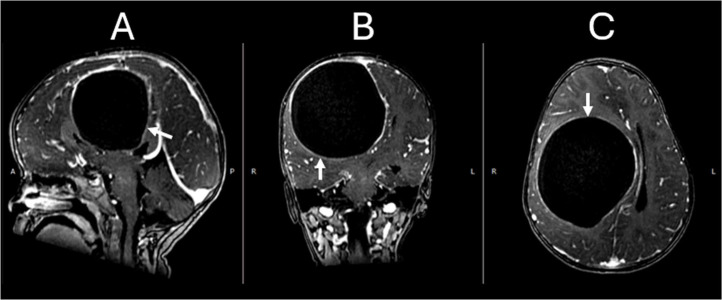
Enhanced T1 Gradient-Echo images in sagittal (A), coronal (B), and axial (C) planes demonstrate a well circumscriebed lesion with a thin wall presenting no significant enhancement (White arrows).

Brain magnetic resonance imaging (MRI) (Figs. 1 and 2), ordered by the primary pediatrician, showed a large intra-axial cystic mass in the right frontoparietal region measuring 9.0×8.0×7.5 centimeters. The lesion caused significant mass effect, displacing the lateral ventricle and midline structures by approximately 30 millimiters, accompanied by subfalcine herniation. Imaging findings were suggestive of a cerebral hydatid cyst, with a brain abscess or tumor considered unlikely due to the mass thin wall, lack of enhancement, and minimal surrounding edema.

The patient was referred and admitted to the Neurosurgery Department for further evaluation and treatment. Laboratory investigations were largely unremarkable, except for mild eosinophilia.

**Fig. 3:**
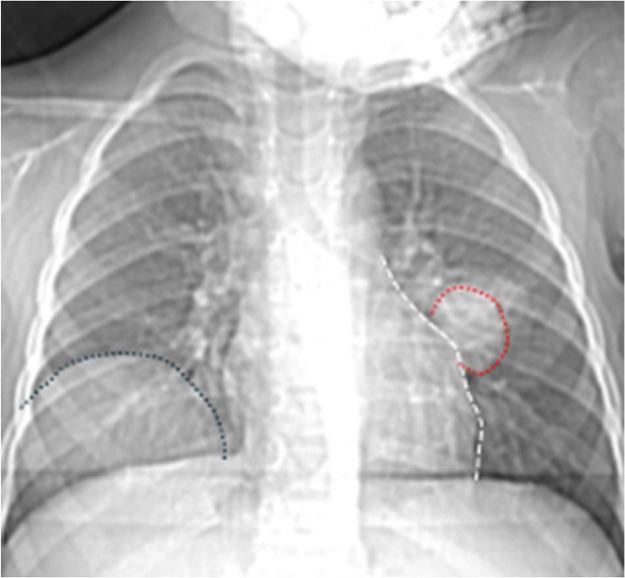
Anteroposterior chest radiograph reveals 2 well-circumscribed, rounded opacities: one located in the right lower lobe (black dotted line) and the other in the left lower lobe (red dotted line). The cardiac silhouette remains clearly demarcated (white dotted line), indicating a negative silhouette sign.

As part of the preoperative assessment, a chest X-ray (Fig. 3) was performed, which revealed 2 rounded opacities of varying sizes in both lung fields. These radiographic findings raised suspicion for cystic lesions, prompting further imaging.

**Fig. 4:**
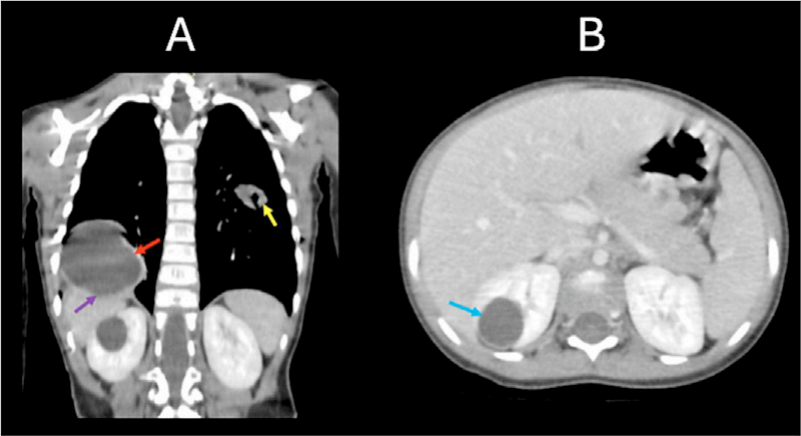
Contrast-enhanced CT scan in the portal venous phase. (A) Coronal reconstruction at the thoracic level demonstrates 2 cystic lesions: a well-defined, round, fluid-filled cyst in the right lung (red arrow), causing inferior displacement of the diaphragm and indentation of the hepatic contour (purple arrow); and an oval-shaped, cavitary lesion with internal air lucency in the left lung (yellow arrow), suggestive of partial cyst rupture. (A & B) Coronal (A) and axial (B) views also reveal a well-circumscribed, round cystic lesion in the upper pole of the right kidney (blue arrows), with homogenous fluid content and a thin, regular wall.

**Fig. 5:**
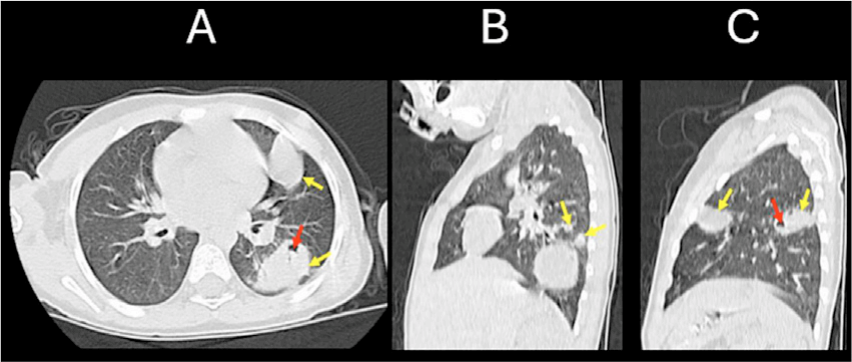
CT scan of the chest in lung window settings. Multiplanar views including axial (A), right lung sagittal (B), and left lung sagittal (C) images demonstrate multiple well-defined, thin-walled cystic lesions distributed across both lungs (yellow arrows). The left lung contains an excavated cyst with internal air-fluid level (red arrow), indicative of partial cyst rupture or bronchial communication.

**Fig. 6:**
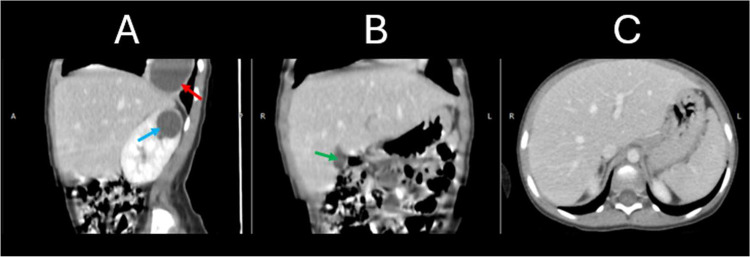
Contrast-enhanced CT scan in the portal venous phase. Multiplanar views including sagittal (A), coronal (B), and axial (C) reconstructions demonstrate no evidence of cystic lesions within the hepatic parenchyma. The gallbladder is visualized with normal morphology (green arrow). A well-defined supra-diaphragmatic cystic lesion is noted in the lower right thoracic cavity (red arrow), and a round, fluid-attenuation cyst is visible in the upper pole of the right kidney (blue arrow).

A thoraco-abdomino-pelvic computed tomography (CT) scan (Figs. 4, Fig. 5, Fig. 6 and 6) was performed as part of the diagnostic workup to evaluate additional cystic lesions. The CT scan revealed multiple cystic lesions of varying sizes located in different segments of both pulmonary fields (the biggest one was located in the lower right lobe measuring 4.0×5.0×4.5 centimeters) including one lesion communicating with a segmental bronchus measuring 2.0×2.8×1.5 centimeters; in addition to a cystic formation at the upper pole of the right kidney. These lesions were characterized by their spherical or oval shape, fluid content, highly suggestive of hydatid cysts given the patient's clinical context.

**Fig. 7:**
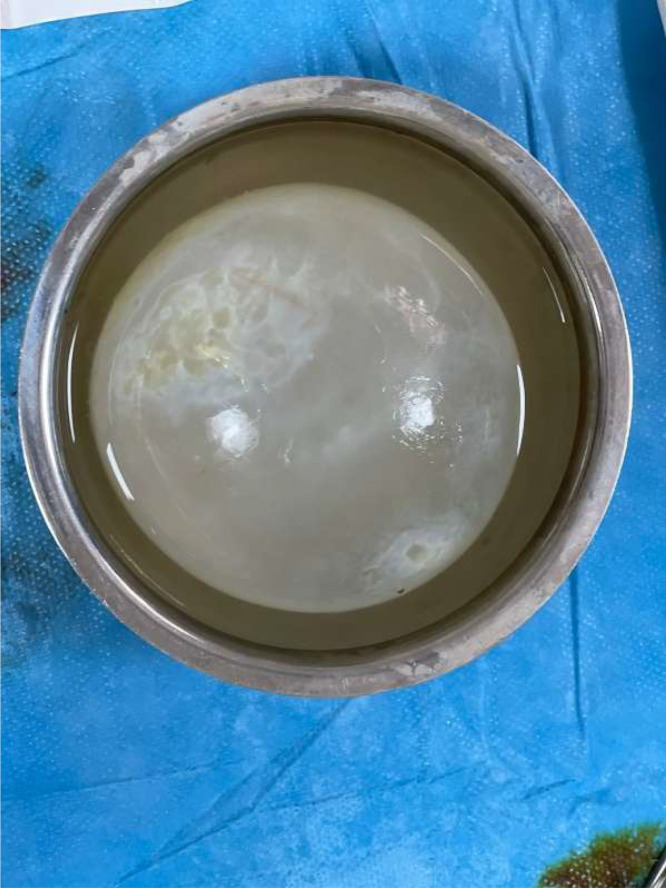
Gross specimen of the excised cerebral cyst.

The surgical excision of the cerebral mass was performed by the Neurosurgery team. The cyst appears as a large, well-circumscribed, spherical structure with a smooth, translucent outer wall. It is immersed in preservation fluid and shows a glistening thin membrane (Fig. 7).

Histopathological analysis of the excised specimen revealed scolices of *Echinococcus granulosus*, confirming the diagnosis of a cerebral hydatid cyst.

The patient was admitted to the Intensive Care Unit under monitoring for post-operative care; multidisciplinary consultations discussed the next steps in managing the remaining pulmonary and renal lesions. She was discharged 48 hours later on albendazole 400 milligrams twice a day, and a 2-month follow-up was planned to assess for additional surgery.

## Discussion

This case represents a rare presentation of pediatric hydatid disease, characterized by simultaneous involvement of the brain, lungs, and kidneys in a 2-year and 3-month-old child, with complete absence of hepatic and splenic lesions. In typical cystic echinococcosis, the liver (60%-70%) and lungs (20%-30%) are the most affected organs, especially in children, due to their filtering role in the portal and systemic circulations [6]. The bypass of hepatic filtration, leading to direct systemic dissemination, has been hypothesized in very few pediatric reports and may involve aberrant circulatory pathways or lymphatic spread [5,7].

In our case, the age of onset—just over 2 years—is particularly remarkable, considering that most disseminated hydatid infections with CNS involvement are diagnosed in children above 5 years of age due to the slow-growing nature of the cysts [8,9]. The child described by Shabbir et al. with disseminated disease involving the brain, lungs, liver, and abdomen was 7 years old [4]. This makes our patient one of the youngest documented with non-hepatic, multiorgan hydatid disease.

The neurological presentation in our patient, with focal deficits and seizures, aligns with known cerebral hydatidosis manifestations in children. Cerebral hydatid cysts are rare, accounting for less than 2% of cases, and are generally supratentorial and solitary [8,10]. Our patient’s MRI revealed a large right frontoparietal cystic lesion with significant mass effect and subfalcine herniation, which is consistent with imaging characteristics previously described in literature [7].

The pulmonary and renal involvement in our patient was discovered through a TAP CT scan. Pulmonary cysts are expected in pediatric echinococcosis, but renal hydatid disease is rare, seen in only 2%-4% of cases, and often incidental unless symptomatic [11]. Following a systematic literature review, the simultaneous detection of renal, cerebral, and pulmonary hydatidosis in a patient this young—and notably, without liver or spleen involvement—has not been previously documented in reports, including the 10-year pediatric series by Şişmanlar Eyüboğlu et al. [8], the reports by Said et al. [5] and the ones from Shabbir et al. [4].

From a radiological standpoint, this case underscores the essential role of multimodal imaging. MRI provides superior soft tissue contrast for intracranial lesions, particularly in delineating perilesional edema and mass effect, while CT remains optimal for screening thoracoabdominal hydatid disease. [6].

Finally, histopathologic analysis also helped exclude mimics such as pyogenic abscesses or cystic tumors, further supporting the diagnosis of primary cerebral echinococcosis. This aligns with previously reported surgical outcomes emphasizing the importance of complete cyst excision and tissue diagnosis [3,8].

To better contextualize our patient’s presentation within the existing pediatric literature on hydatid disease, we compiled a comparative overview of reported cases and series detailing multiple organ involvement, patient age ranges, and the presence or absence of liver localization. The table below summarizes key clinical characteristics from selected case reports and pediatric cohorts referenced in this study.

In summary, as illustrated in Table 1, our case represents the youngest reported patient with multiorgan hydatidosis without hepatic involvement, based on the reviewed pediatric series and case reports. It emphasizes the need for comprehensive imaging in endemic areas even when liver imaging is negative, and highlights how early presentation, unusual dissemination, and absence of typical involvement can challenge conventional diagnostic pathways.

**Table 1:** Comparative table of multiorgan pediatric hydatid disease cases.

Author(s) & year	Patient age	Organs involved	Multiorgan dissemination	Liver involvement	Diagnostic modality	Histopathology confirmation	Notable remarks
Our Case (2025)	2 yrs 3 mo	Brain, Lungs, Kidney	Yes	No	MRI + CT	Yes	Youngest reported with multiorgan non-hepatic hydatidosis
Mirlohi et al. [11]	10 yrs	Lungs, Kidney	Yes	No	US + CT	Yes	Multiorgan dissemination with no liver involvement
Shabbir et al., [4]	7 yrs	Brain, Lungs, Liver, Abdomen	Yes	Yes	US + CT	Yes	Disseminated case with liver involvement
Olmez et al., [7]	5 yrs	Liver, Lung, Spleen, Kidney	Yes	Yes	CT	Yes	Disseminated case with liver involvement
Gupta et al., [10]	12 yrs	Liver, Lung, Spleen, Kidney	Yes	Yes	US + CT	Yes	Multiple Organs dissemination with liver involvement
Said et al., [5]	2-16 yrs	Liver, Lungs, Brain	Yes	Yes	CT + MRI	Yes	Series of pediatric cases, liver dominant
Şişmanlar Eyüboğlu et al, [8]	1-16 yrs	Liver, Lungs, Other	Yes	Yes	CT	Yes	10-year pediatric cohort
Kaya et al., [12]	4 yrs	Liver, Lungs Kidney	Yes	Yes	US + CT	Yes	Multiorgan dissemination with multiple cysts

## Conclusion

This report highlights an uncommonly reported presentation of pediatric hydatid disease involving simultaneous cerebral, pulmonary, and renal cysts in a very young child, notably without hepatic or splenic involvement. The absence of liver and spleen localization challenges the classical understanding of cystic echinococcosis dissemination and underscores the importance of considering hydatid disease in unusual organ distributions, especially in endemic areas. Early multimodal imaging, including brain MRI and thoraco-abdomino-pelvic CT, proved crucial in detecting the full extent of the disease. This case reinforces the need for vigilance in endemic areas and consideration of hydatid disease even when liver imaging is negative and CNS involvement predominates. While surgical excision remains the definitive method for diagnosis, inoperable cases may require serologic or imaging-based confirmation strategies. This report contributes valuable insight into the variable clinical patterns of hydatid disease in pediatric populations and emphasizes the need for comprehensive evaluation even when typical hepatic cysts are absent.

## Ethical statement and consent for publication

The authors stated that an informed consent was obtained from the patient.

## Data sharing statement

Data supporting the findings and conclusions are available upon request from the corresponding author.

## Author contributions

All authors were involved in writing and critically revising the article. All authors approve the final version of the article.

## Patient consent

Written informed consent was obtained from the patient for publication of this case report and accompanying images.
